# The ICMJE and URM: Providing Independent Advice for the Conduct of Biomedical Research and Publication

**DOI:** 10.4103/0973-1229.32145

**Published:** 2007

**Authors:** Martin B Van der Weyden

**Affiliations:** *Editor, Medical Journal of Australia and Member of the International Committee of Medical Journal Editors (ICMJE), Australia*

**Keywords:** *ICMJE*, *URM*, *Trial Registries*, *Vancouver Group*, *Biomedical Research*, *Issues In Ethical Research*, *Editorial Freedom*, *Industry Influence*

## Abstract

The International Committee of Medical Journal Editors (ICMJE) is a working group of editors of selected medical journals that meets annually. Founded in Vancouver, Canada, in 1978, it currently consists of 11 member journals and a representative of the US National Library of Medicine. The major purpose of the Committee is to address and provide guidance for the conduct and publishing of biomedical research and the ethical tenets underpinning these activities. This advice is detailed in the Committee's Uniform Requirements for Manuscripts Submitted to Biomedical Journals: Writing and Editing for Biomedical Publication (URM).

Recently, the ICMJE has adopted an interventionist role to ensure transparency of conflict of interest revelations in the conduct and publication of industry supported research. It also pursues a policy for the lodgement with trial registries of specified details of Phase III clinical trials. Failure to comply would jeopardise publication of trial outcomes in ICMJE member journals. This policy has resulted in the coming on stream of trial registries, international agreement on trial minimal datasets and compliance with trial registration requirements.

## Introduction

Traditionally academic medicine encompasses patient care, teaching and research. Until recently all were of equal value. But with the explosive growth of biomedical research since the 1960s, research now rules supreme (Ludmerer, 1999). The modern juggernaut of biomedical research is fuelled by many factors, including the realisation by governments of the economic potential of research (Bayh -Dole Act, 1980; Tattam, 1999), the commercialisation of research through the interaction of investigators and their institutions with corporations and venture capitalists (Bok, 2003; Krimky, 2003) and a change in the attitude of academia towards industry (Angell, 2000). All these developments have had direct influences on the conduct and reporting of biomedical research.

Research is a messy business, according to Frank Davidoff, Emeritus editor of the *Annals of Internal Medicine,* who recently commented that, “researchers function in an uncertain universe where they are required to continually break the mould, wallow in data (and) filter out tiny signals from a mass of information”. He also argues that science does not exist until it is published and until, “a piece of scientific work has reached a state of orderliness completeness and coherence suitable for public release and (is) captured in a stable medium that can be efficiently distributed and easily retrieved” (Davidoff, 2000). In short, the researcher moves from the chaotic world of research to the orderly world of publishing in which the quality filters of editors, peer reviewers and manuscript editors help the researcher create a highly structured scientific record. Essential to this is a framework to guide the preparation and submission of research manuscripts and advice on ethical and other issues involved in the process of publishing. One such noteworthy advice is as conveyed in the Uniform Requirements for Manuscripts submitted to biomedical journals [URM], first published in 1979 by the International Committee of Medical Journal Editors [ICMJE] (Huth and Case, 2004).

## The International Committee of Medical Journal Editors

The ICMJE evolved from a small group of medical editors that met in 1978 at Vancouver, Canada, to establish uniform guidelines for the format of manuscripts submitted to their journals. Known initially as the International Steering Committee, the group later adopted the title ICMJE. Reflecting the location of its inaugural meeting it is also known as the Vancouver Group (Huth and Case, 2004). The editors who participated in the first meeting were from the *American Review of Respiratory Disease (Am Rev of Res Dis), Annals of Internal Medicine (Ann In Med), The Lancet, Journal of the American Medical Association (JAMA)* and the *New England Journal of Medicine (N Eng J Med).* Along with them, some individuals who played a major role in the establishment of the Committee were Edward J. Huth [*Ann Int Med*], Stephen Lock [*BMJ*], Jock F. Murray [*Am Rev Res Dis*] and Theresa Southgate [*JAMA*] (Huth and Case, 2004). It is humbling to reflect that this eminent body has its roots in a simple request a decade earlier by a medical secretary at the University of Washington Medical School in Seattle who grew tired of re-typing references in different formats when rejected manuscripts were submitted to journals. Those were the days of the cumbersome typewriter! She wrote to the editors of the *Ann Int Med, N Eng J Med and JAMA*. Two years later, these and eighteen other journals agreed to use the format of *Index Medicus* specified by the National Library of Medicine (Huth and Case, 2004).

Today, the ICMJE is a working group of editors of selected general medical journals and is not an open membership organization. Current participating journals, in alphabetical order, are the *Ann Int Med, BMJ, Canadian Medical Association Journal, Croatian Medical Journal, Dutch Medical Journal, JAMA, Journal of the Danish Medical Association, Journal of the Swedish Medical Association, The Lancet, Medical Journal of Australia, N Eng J Med, New Zealand Journal of Medicine* and the *US National Library of Medicine.* Each journal serving on the Committee is restricted to one representative. The exception is the journal that provides the committee's secretariat functions (currently, the *Ann Int Med*). This deliberate restriction in the number of committee members reflects a desire to keep the committee small and to ensure comprehensive discussion. In its nearly 30-year history, the ICMJE has successfully eluded a formal structure and the trappings of bureaucracy. It has no constitution, no executive, no chair and is self-funded; and, despite its inherent authority, it has no jurisdiction over other journals. The hosting journal of the annual meeting is decided informally at the previous year's meeting and the host is free to invite guests, some of who eventually continue as members. At its 2006 meeting in Oslo, Norway, the group agreed to invite a general medical journal from the developing world as a guest to the 2007 meeting in Sydney, Australia.

## The Uniform Requirements for Manuscripts Submitted to Biomedical Journals (URM)

Since the first version of the URM in 1979, there have been four further editions and numerous revisions (Huth and Case, 2004). In 2000, the ICMJE decided to maintain the URM as an electronic document accessible at a central website (www.icmje.org). This approach has obviated the need to identify editions and the latest version of the URM was updated in 2006.

The current URM represents the cumulative contributions of ICMJE members over nearly thirty years. It outlines guidelines for the construct of manuscripts along with issues related to the ethical conduct and reporting of research and issues governing editorial and publishing functions (International Committee of Medical Journal Editors, 2006) [Table 1].

**Table 1:** Outline of the Uniform Requirements for Manuscripts for Submission to Biomedical Journals

Detailed advice related to:

**Manuscript preparation and submission**
The manuscript's title page, notification of conflict of interest, abstract format and key words, the manuscript construct [introduction, methods, results and discussion], references style and guidelines for tables, illustrations and their legends, measurement units, abbreviations and symbols and final procedures to be followed in submitting the manuscript.
**Ethical Considerations in the conduct and reporting of research**
Authorship and contributorship, the role of the editor and editorial freedom, methods of peer review and obligations of peer reviewers, conflict of interest as applicable to authors, editors, journal staff and peer reviewers and conflict of interest arising from research support, privacy and confidentiality requirements covering patient and study participants, authors and peer reviewers and provision for the protection of human subjects and animals in research.
**Publication and editorial issues**
Obligation to publish negative studies, processes to follow for corrections, retractions and expression of concerns, copyright, overlapping publications, duplicate submission, redundant publication, secondary publication, competing manuscripts based on the same study but with differing analysis or interpretation and or difference in reported methods, advice on journal correspondence columns, guidelines covering supplements, theme issues and special services, electronic publication, advertising, interrelationship between medical journal and the general media and finally the obligation to register clinical trials.

In the early years, the ICMJE focused on addressing details of manuscript format such as how to present the title page, abstract, the body of the manuscript (introduction, methods, results and discussion-the IMRAD format) and the references. More recently, these guidelines have included advice on Supplements, Theme Issues and requirements for specific study designs such as randomised controlled trials, studies of diagnostic accuracy and systematic reviews and meta-analysis.

Before long, the ICMJE shifted its focus to both practical and ethical issues facing authors and editors, such as authorship, advertising, conflict of interest, duplicate (overlapping) publication and research misconduct, which recently reached new heights with the high profile instances of fraudulent research in Korea and Sweden (Parry, 2006; Eaton, 2006). Editorial freedom featured early on the ICMJE agenda with the clear advice that editors should have complete control over the content of their respective journals. With the recent summary  sacking of editors of the *CMAJ*, John Hoey and Anne Mary Todkill (Van Der Weyden, 2006; Singh and Singh, 2006), the ICMJE reaffirmed its endorsement of the WAME definition of editorial freedom (The World Association of Medical Editors, 2006) - an act that represents an unusual direction for the Committee, as in the past it has only endorsed its own statements.

## The Growing Influence of Industry on Biomedical Research

### Sponsorship, Authorship and Accountability

In 2003, funding of biomedical research in the United States reached the astronomical figure of $ 94.3 billion. Industry accounted for 57% of this funding, the three major contributors being pharmaceutical, biotechnology and medical devices firms (Moses III, *et al.*, 2005). With this level of financial investment, it should come as no surprise that the purpose of biomedical research has insidiously slipped from being for the public good to being for the good of researchers, their institutions and the funding corporations. Indeed, academic success is now in part determined by researchers’ involvement in industry as advisors, consultants, board members and participants in industry speaker bureaus. Underpinning all this is the capacity to secure ongoing research funding, which is largely determined by publication performance. Indeed to the adage of, “publish or perish”, might well be added, “publish in high impact journals and prosper personally and professionally.”

**Figure 1:**
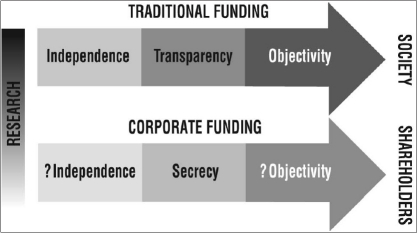
Clash of research ideals with corporate funding of research

The involvement of industry in biomedical research is one of the reasons for the success of biomedical research and its beneficial contributions to clinical practice. After all, industry has an imperative to translate research into tangible products for the healthcare market. But it also has a down side - a potential erosion of research ideals such as transparency and objectivity [Figure 1].

Negative effects such as biased interpretation (Friedman and Richter, 2004), restrictions on publication of results (Whittingham, *et al.*, 2004) and a culture of commercial secrecy (Giles, 2006), are particular prevalent in industry sponsored trials of drugs.

The average cost of developing a new drug is estimated to be US$ 300–600 million (Makien, 1999) and for each day's delay in gaining drug approval by the US Food and Drug Administration, it costs the manufacture about US $1.3 million (Bodenheimer, 2000). Furthermore, pharmaceutical sponsorship of clinical trials is big business: in 2003 it amounted to a staggering US $14.3 billion (Moses III, *et al.*, 2005). With this level of investment, it is no surprise that timeliness is of utmost importance and can be best achieved by control of trial processes - study design; data collection, analysis and interpretation; who should be authors: whether members of the trial publication committee, employees of the sponsoring company or professional scientific writers; and whether to proceed or not with publication or what should be published (Friedman and Richter, 2004; Whittingham, *et al.*, 2004; Giles, 2006; Bodenheimer, 2000). Attempts by sponsoring companies to control research and the publication through outsourcing to commercial contract research organizations [CROs] and site - management organizations [SMOs] in the 1990s (Bodenheimer, 2000) prompted the ICMJE, in 2001, to publish simultaneously in all its members′ journals a committee written statement, “Sponsorship Authorship and Accountability” (Davidoff *et al.*, 2001). This act broke new ground for the ICMJE as it moved from a passive advisory group to one pursuing an active interventionist role.

The ICMJE statement detailed a set of revised ethical principles applicable to authors, peer reviewers and journal staff and guidelines for managing conflict of interest in the conduct and publication of clinical trials. It noted that, “Authorship means both accountability and independence. A submitted manuscript is the intellectual property of authors, not the study sponsor. We will not review or publish articles based on studies that are conducted under conditions that allow the sponsor to have sole control of the data or to withhold publication. We encourage investigators to use the revised ICMJE requirements on publication ethics to guide negotiation of research contracts. Those contracts should give the researcher a substantial say in trial design, access to raw data, responsibility for data analysis and interpretation and the right to publish- the hallmarks of scholarly independence and ultimately academic freedom” (Davidoff, *et al.*, 2001).

### Clinical Trail Registration

More recently, the ICMJE turned it attention to the registration of clinical trials. The need for trial registration is self-evident - it is an antidote to the poisoning of clinical evidence through the failure to publish interventional trials with negative or adverse outcomes. This failure to publish has serious consequences for clinical practice and public trust in clinical research. Indeed the need for publication of all interventional trial and transparency about trial conduct has been argued over in the medical literature for more than thirty years (dChalmers, 1977; Simes, 1986; Chalmers, 1990; Dickersin and Rennie, 2003).

In September 2004, the ICMJE announced that journals represented on the Committee would not publish results of any ongoing interventional trials [phase III] that had not been registered by September 2005 with an appropriate trial register (DeAngelis *et al.*, 2004). The latter was defined as owned and operated by a not-for-profit organization; that requires lodgement of a minimal trial dataset and that the registry be electronically freely accessible to any interested party. The Committee recommended a minimal dataset and subsequently it endorsed a WHO mandated dataset of 20 fields (DeAngelis, *et al.* (2005). In 2004, the only registry that satisfied the ICMJE criteria was ClinicalTrials.gov (http://www.clinicaltrials.gov/) of the US Library of Medicine at Bethesda, Marylands. In December 2005, Zarin and her colleagues from that registry reported on the compliance of trial sponsors with ICMJE requirements before and after its September 2005 deadline for trial registration (Zarin *et al.*, 2005). The number of trials registered at ClinicalTrials.gov increased by 79% (13, 153 to 22, 714) and the percentage of trials registered with non-specific identification of the intervention decreased from 12% to 2%. With complete compliance in academically driven trials and variability in commercial sponsored trials, two major pharmaceutical firms continued to use meaningless interventional designations in 11 to 22 percent of registrations (Zarin *et al.*, 2005). Subsequently, the fulfilment by the pharmaceutical industry of the trial intervention and outcome fields has improved tremendously (Drazen *et al.*, 2007) and it is uncommon for ICMJE journals to receive submissions of unregistered trials (Personal Communication ICMJE members, Jan 2007). This represents a remarkable change in culture.

Over the last two years, the World Health Organisation (WHO), through its International Clinical Trial Registry Platform (ICTRP), has addressed an internationally acceptable trial registration data set and which clinical trials should be registered. In May 2006, the ICTRP formally announced its 20 - items trial registration data set [Table 2] and called for registration of all interventional trials, including early phase uncontrolled trials (phase 1), along with full public disclosure of all registration data items at the time of registration and before recruitment of the first participants (Sim *et al.*, 2006).

**Table 2:** The International Clinical Trial Registry Platform's 20-item trial registration dataset

1	Primary register and trial registration number
2	Date of registration in primary register
3	Secondary identification number(s)
4	Source(s) of money or material support
5	Primary sponsor
6	Secondary sponsor
7	Contact for public queries
8	Contact for scientific inquiries
9	Public title
10	Scientific article
11	Countries of recruitment
12	Health condition(s) or problem(s) studied
13	Intervention
14	Key inclusion and exclusion criteria
15	Study type
16	Date of first enrolment
17	Target sample size
18	Recruitment Status
19	Primary outcome(s)
20	Secondary outcomes

Considerable progress has been made since the ICMJE's foray into trial registration. There are now four recognised trail registries world wide: in Australia (http://www.actr.org.au), Japan (http://www.umin.ac.jp/ctr/index/htm), Europe (http://isrctn.org) and the US (http://www.clinicaltrials.gov). There is even discussion on posting trial outcome data on an independent results database instead of publishing papers in journals (Horton, 2006).

### Influence of the ICMJE

At its inception, the ICMJE was a small group of major Anglophone journals. It has grown to be more representative although some would argue that it has northern hemisphere bias. But in the words of Edward Huth and Kathleen Case in their account of the first twenty five years of the ICMJE, “….a small group of decision makers unhampered by bureaucracy can accomplish much.” (Huth and Case, 2004). And so it has. It has provided leadership in guidance for the publication and conduct of biomedical research and will continue to do so. It unashamedly insists that biomedical research be ethical, transparent and objective and it encourages the research and publishing communities to identify issues that require the Committee's attention.

## Questions That This Paper Raises

- Are there better ways to develop global guidance for conduct of biomedical research and publication?
- Should there be uniformity in this guidance? There are many bodies proffering advice, including the ICMJE, WAME, CBE and EASE.
- Should Journals be graded for compliance with the Uniform Requirements for Manuscript submitted to Biomedical Journals?
- Will commercialisation of research result in progressive harm to the integrity of biomedical research and publication? Who should be the guardian of this integrity?
- Will publication quality suffer because of the extensive number of biomedical journals with a resultant dilution of editorial expertise, pressure on finite numbers of peer reviewers and acceptance of inferior articles to fill space?

## About the Author

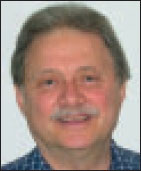



Martin Van Der Weyden, has been Editor of The Medical Journal of Australia since 1995 and Chief Executive of the Australasian Medical Publishing Company since 1996. A graduate of Sydney University, Martin has had a varied career in academic and clinical medicine and hospital administration. He was a Merck Sharpe and Dohme International Fellow in Clinical Pharmacology and a National Science Foundation Fellow at Duke University Medical Centre, North Carolina. On return to Monash Medical School at Alfred Hospital, Melbourne, he was appointed as an NHMRC Research Fellow and, subsequently, Associate Professor of Medicine and Professor of Haematology. At the Alfred, he was a senior visiting physician and head of the Haematology Services. Not satisfied with these challenges he was recruited into administration as Chief of Investigative Medicine before joining The Medical Journal of Australia. He has been a member of the ICMJE since 1995. He has published more than 200 articles in clinical research and on editorial or medical professional issues.
